# New Graft Choices for ACL Reconstruction: Update Article

**DOI:** 10.1055/s-0044-1779335

**Published:** 2024-04-23

**Authors:** Paulo Henrique Schmidt Lara, João Victor Novaretti, Gilvan Rodrigues da Silva Nunes, Moises Cohen, Leonardo Addêo Ramos

**Affiliations:** 1 Centro de Traumatologia do Esporte, Escola Paulista de Medicina, Universidade Federal de São Paulo, São Paulo, SP, Brasil; 2 Departamento de Ortopedia e Traumatologia, Escola Paulista de Medicina, Universidade Federal de São Paulo, São Paulo, SP, Brasil

**Keywords:** anterior cruciate ligament, grafting, knee

## Abstract

Reconstruction of the anterior cruciate ligament (ACL) is a common procedure for injuries to this ligament, especially in athletes. There are different types of grafts used, and the choice depends on several factors. Autologous grafts, from the patients themselves, are the most common option, with rapid incorporation and a lower failure rate. Allografts from donors have their role in specific cases. Synthetic grafts, used in the 1980s, have advantages such as the absence of morbidity at the donor site, but studies have shown long-term complications. Hybrid grafts, combining autologous grafts and allografts, have gained interest, allowing a larger diameter and reducing morbidity. Peroneus longus tendon autograft has received attention, with positive results, good knee function and less hypotrophy of the thigh at the donor site. Autologous quadriceps tendon graft has gained popularity, with results comparable to patellar and flexor tendon grafts, lower morbidity at the donor site and a lower rate of re-rupture. The choice of graft has evolved, with autologous flexor grafts being preferred for less active patients and patellar grafts with bone fragments for high-performance athletes. Allografts, synthetic and hybrid grafts have their role in specific circumstances. The choice must be based on scientific evidence, considering advantages and disadvantages. ACL reconstruction is a complex procedure that requires individual considerations to select the most appropriate graft.

## Introduction

**Fig. 1:**
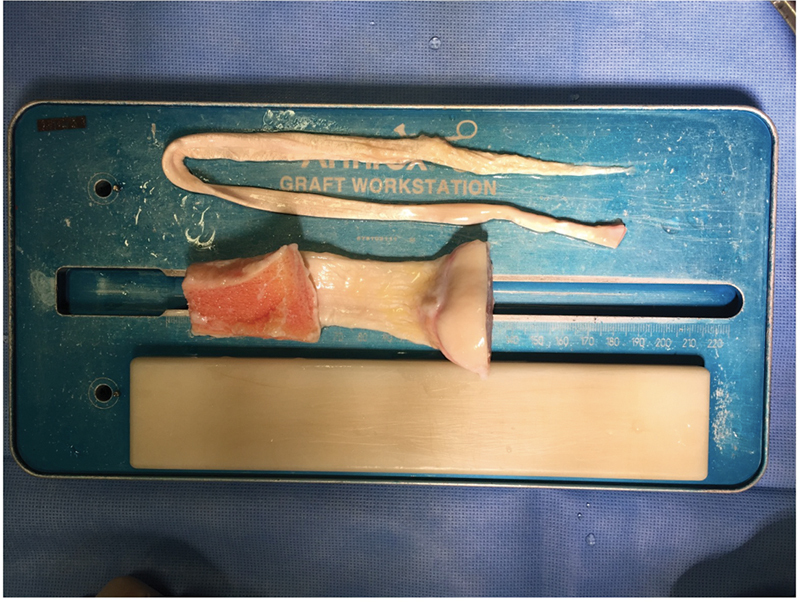
Photo of allografts ready to be prepared. Grafts with a bone part (Patellar) and without bone parts (Tibial Posterior) are observed.

Anterior cruciate ligament (ACL) rupture is a common injury in the general population, with an incidence of up to 75 per 100,000 people per year, 1 particularly in active individuals involved in contact sports. Although a reconstructed ACL does not completely restore the original structure or biomechanical properties of the native ACL, 2 the graft used for reconstruction must not only have structural and mechanical properties that resemble those of the native ligament, it must also exhibit minimal antigenicity and sufficient innate biological potential to incorporate into the host's bone. When selecting graft types, there are several considerations: autograft versus allograft and soft tissue-only grafts versus grafts with bone fragments. Examples of allografts are shown in Fig. 1 .

**Fig. 2:**
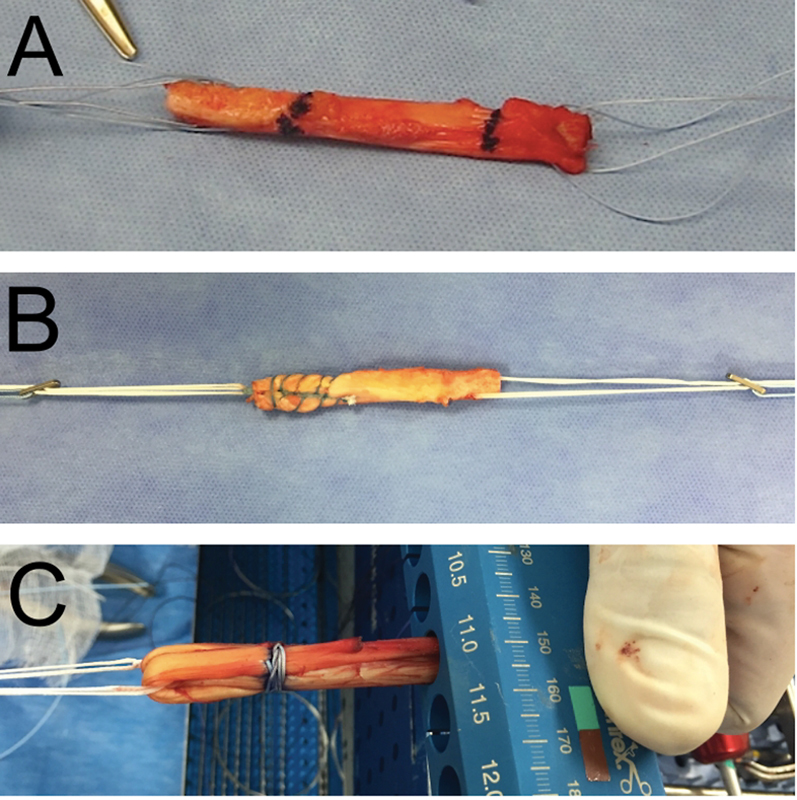
Different types of grafts: (A) patellar tendon graft, (B) quadriceps tendon graft with bone plug, (C) sextuple flexor graft.

The commonly used autografts are: patellar with bone fragment, knee flexors, quadriceps (with or without patellar bone fragment); Among allografts, additional options include anterior and posterior tibial, peroneal, and calcaneal. 3 4 5 6 7 In Fig. 2 different types of grafts are demonstrated.

Optimal graft selection depends not only on the properties of the graft, but mainly on the patient's characteristics and expectations.

## Autograft versus Allograft

All allografts demonstrated slower rates of incorporation compared to autografts, as well as a higher failure rate of approximately 25% in the active population (43 versus 75%). 8 Current evidence suggests the use of allografts in specific circumstances such as multiligament knee reconstructions, inadequate autograft tissue, or in older, less active populations. 9 The theoretical advantages of allografts are: elimination of donor site morbidity, less pain, shorter surgical and rehabilitation times and better cosmetic results. 10 Krych et al. 11 reported a fivefold higher risk of rerupture in cases that used an allograft. When excluding irradiated and chemically processed grafts, there was no difference in rerupture rate; however, their systematic review included only 6 studies. Kraeutler et al. 12 demonstrated similar results with a risk of rerupture approximately 3 times higher in the allograft group (12.7% vs. 4.3%). They also demonstrated increased knee laxity, and worse results in the single-leg hop test and subjective satisfaction. 12

The prospective cohort study carried out by Kaeding et al. 13 evaluated the number of variables to determine predictors of graft rupture in the 2 years after reconstruction. Allograft use and young age significantly increased the risk of graft rupture. 13 Other recent studies have found increased rates of graft rupture in patients who received allografts and had a high level of postoperative activity. 14 15 16

In laboratory and clinical studies, autografts show better results than irradiated and processed allografts. 17 Allografts may be considered for less active patients who are willing to accept the increased risk of graft failure. 17

In an outpatient surgery setting, the autograft group had a significantly lower bill than the allograft group. 18 The decrease in surgical time did not compensate for the cost of the allograft (around $1,000). 18

Regarding graft processing, it is believed that sterilization techniques alter biomechanical properties and the more a graft is processed, the worse its performance. 17 Park et al. 19 performed a systematic review of irradiated versus non-irradiated allografts with at least two years of follow-up, demonstrating worse functional scores in the irradiated group, decreased stability for Lachman, pivot-shift and KT-1000 testing, and increased risk of revision. The study by Tian et al. 20 demonstrated similar results. Allograft sterilization techniques alter the mechanical properties of allografts and are categorized into radiation or ethylene oxide. The extent of change in mechanical properties is dependent on the irradiation exposure dose. 7

Farago et al. 21 reviewed 29 years of articles that evaluated the impact of sterilization techniques on tendons. Review results support that the technique with the greatest biomechanical preservation was freezing followed by radiation at 14.8-28.5 kGy. 21 However, allograft failure is not solely attributed to sterilization techniques, whereas allograft failure rates still remain high when comparing fresh allografts to autografts. 21

## Synthetic Grafts

Synthetic grafts were initially used in the 1980s as an option, offering advantages such as the absence of donor site morbidity, shorter surgical time, and reduced risk of disease transmission. 22 23 24 And potentially the possibility of an earlier return to sports. 22 23 24 However, early studies reported satisfactory short-term results, but in the medium and long term, there were complications such as an immune response, foreign body synovitis, tunnel osteolysis, fractures of the femur and tibia near the tunnels, and late graft failure. 22 23 24 25 26 This resulted in a decline in the use of synthetic grafts, but there is currently renewed interest in a new generation of artificial grafts that have shown favorable results when used in special circumstances, such as in the older population. 24 25 It has gained some popularity among athletes recently due to the potential for immediate graft stability, faster rehabilitation, and a quicker return to sports. 27 A systematic review conducted by Machotka et al. 28 on the Ligament Advanced Reinforcement System (LARS) recommended caution when considering the use of synthetic grafts, as more studies are needed. In the study by Bianchi et al. 29 comparing LARS and knee flexor grafts, the LARS group demonstrated greater stability, and no patient required revision surgery. LARS can be considered in patients who require a rapid recovery, while being aware of the risk of graft failure and iatrogenic osteoarthritis. 30 31

## Hybrid Graft

It consists of a combination of auto and allografts and was initially described in 2015. 32 These grafts, typically formed from a combination of autologous flexor graft and soft tissue allograft, have gained interest from orthopedists for use in ACL reconstructions. 33 They are generally used in cases of small size of flexor grafts. 32 When planning to use hybrid grafts, only the semitendinosus graft can be removed instead of the semitendinosus and gracilis. Therefore, the use of hybrid grafts can reduce postoperative morbidity at the donor site. 33 A non-irradiated posterior tibial or peroneus longus allograft is generally used. In addition to the benefit of lower donor site morbidity, hybrid grafts also allow the graft to have a larger diameter than the semitendinosus/gracilis autograft, which may reduce the risk of postoperative failure. Therefore, hybrid grafts may be an option for older patients. 33

## Peroneus Longus Tendon Autologous Graft

**Fig. 3:**
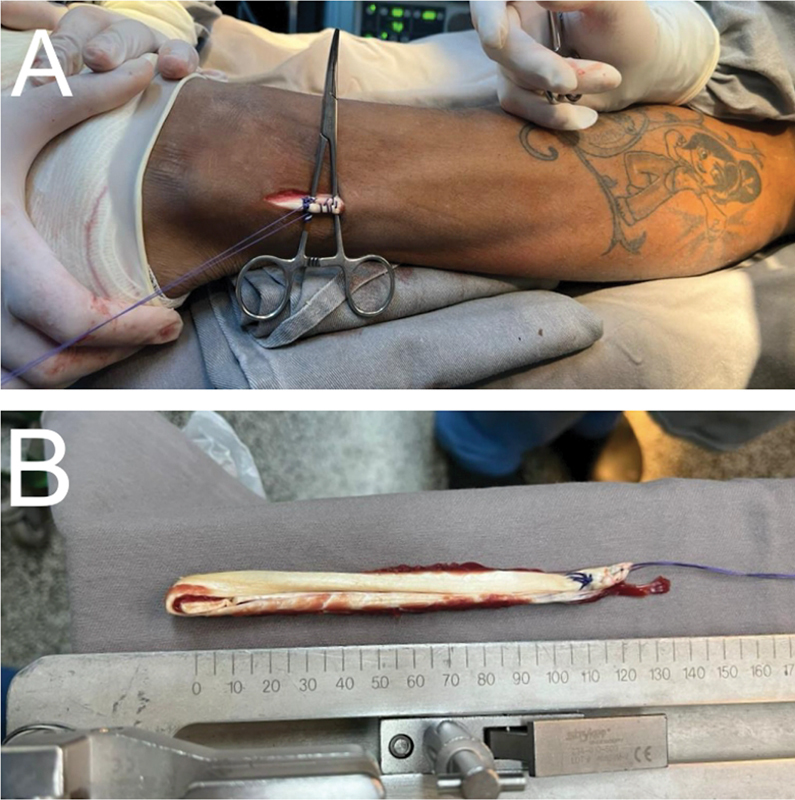
Peroneus brevis graft: (A) small access route for graft removal with minimal morbidity and (B) peroneus brevis tendon graft ready to be prepared on the table with good length and good thickness.

As an autologous graft, the peroneus longus muscle tendon is an old option, however it has received greater attention in recent years due to its biomechanical properties similar to the native ACL ligament and the hamstring graft.. 34 35 36 Fig. 3 shows the graft and its removal.

More recent studies have evaluated its use in ACL reconstruction as well as postoperative function and donor site morbidity. These studies consistently demonstrate positive results, supporting the viability of autologous peroneus longus tendon graft as a good graft option in reconstructions. 37 38 39 40

In terms of functional results, patients undergoing ACL reconstruction using the peroneus longus tendon autograft achieved excellent scores on several assessment tools, the main ones being the IKDC, Modified Cincinnati, Tegner-Lysholm, AOFAS and FADI scores. 35 41 42

These scores indicate good knee function, ankle stability, and overall patient satisfaction. Functional results comparable to traditional graft options such as autologous hamstring tendon graft were also achieved. 35 43 44 Furthermore, autologous peroneus longus tendon graft has demonstrated advantages over other graft options. It featured a larger graft diameter, which may contribute to improved mechanical properties and stability. 35 36 39 45 It was associated with less thigh hypotrophy, indicating a reduction in muscle loss at the donor site. Donor ankle activity was not compromised as evidenced by positive scores on ankle function assessment tools and jumping tests. 35 36 40

Another factor that makes the peroneus longus muscle tendon an ideal candidate for ACL reconstruction is that it is technically safe and easy to remove. 46 The tendon is superficially located and its position is not difficult to access by adjacent structures, such as the tendons of the hamstring muscle. 46 47

Morbidity at the donor site was minimal, with no significant differences observed in ankle eversion and plantarflexion strength of the first ray between the donor site and the contralateral healthy site. 35 36 These findings suggest that autologous peroneus longus tendon graft does not cause significant morbidity at the donor site. 48

In conclusion, ACL reconstruction using autologous peroneus longus tendon graft is a scientifically supported procedure with favorable results. It offers functional results comparable to traditional graft options such as hamstring tendon and can be used as an alternative to the autologous grafts most commonly used for ACL reconstruction: the patellar tendon and the tendon of the hamstring muscle.

## Autologous Quadricipital Tendon Graft

The autologous quadriceps tendon (QT) graft for ACL reconstruction, despite currently being one of the least used grafts, has seen an increase in popularity in recent years, both for primary reconstructions and revisions. 49 50 QT graft can be used with or without a bone fragment removed from the patella. The advantages of removing the QT graft with a bone fragment are a longer graft and a possible better integration of the bone part of the graft into the tunnel created for the graft. The disadvantages are possible residual pain at the site of removal of the patellar bone block and the risk of fracture of the patella. Despite these differences, a recent systematic review compared the use of the QT graft with or without a bone fragment and showed that both grafts are safe and viable, with comparable clinical results, complications and revision rates. 51 Furthermore, QT graft can be used with partial or full thickness, with no difference between them in a recent systematic review. 52

When compared to other graft options, the QT graft has benefits such as lower morbidity at the donor site (defined as anterior knee pain, difficulty or inability to kneel or both) when compared to the patellar tendon autograft. 53 In a cohort study, ACL reconstruction using a QT graft showed a lower rate of re-rupture when compared to autologous flexor graft. 54 In a recent study with 6,652 ACL reconstructions, cases using QT graft had a lower rate of septic arthritis when compared to cases using flexor grafts, patellar tendon grafts or allografts. 55 Regarding biomechanics, QT graft has an elastic modulus similar to that of the native ACL, which, at least from a theoretical point of view, is positive as it would allow biomechanics closer to the biomechanics of the knee before the injury. 56 57 Meanwhile, both the patellar tendon graft and the flexor graft have a significantly higher modulus of elasticity than that of the native ACL. 57 58 As for the load to failure, QT graft presents a load similar to the load of the flexor graft and a load significantly greater than the loads of the patellar tendon graft and the native ACL. 56 57 58 59 60 Finally, regarding the clinical results of graft rupture rate and patient-reported outcomes, the current literature does not present significant differences when QT graft is compared to patellar tendon and flexor grafts. 61

## Discussion

The study by Arnold et al. 61 demonstrated the result of research conducted over the last 14 meetings of the ACL group regarding the preferred graft type in ACL reconstructions. Over time, the choice of graft type can be divided into 4 phases: dominance of the autologous patellar graft with a bone fragment; dominance of the autologous patellar graft with a bone fragment with an increase in the use of autologous flexor grafts; dominance of autologous flexor grafts with a decrease in the use of autologous patellar grafts with a bone fragment and an increase in allograft use; and finally, dominance of autologous flexor grafts with the maintenance of the levels of flexor graft selection and an increase in autologous quadriceps grafts. Currently, more than 50% of the respondents state that their first choice is autologous flexor grafts, while fewer than 40% use autologous patellar grafts with a bone fragment. Allografts increased in popularity from 2006 to 2012, reaching 12% of choices in 2012. Currently, only 1% of the respondents use allografts as their first choice, and none use allografts with a bone fragment. Autologous quadriceps grafts have increased in frequency of selection since 2014, with a peak of over 10% in 2018. 61

When selecting a graft for a primary ACL reconstruction, we must take into account a series of factors, including age, activity level, previous injuries, among others. Each graft option has its advantages and disadvantages. Patellar and knee flexor autografts are still the most used, but there are other options as shown in the initial part of this article. Allografts, quadriceps autograft and peroneus longus autograft are options and their main advantages and disadvantages are shown in Table 1 .

**Table 1:** Advantages and disadvantages of the aforementioned grafts

Types of graft	Advantages	Disadvantages
Allograft	- Absence of donor site morbidity - Choice of graft size - Shorter surgical time - Better cosmetic result	- High cost - Low availability - Risk of disease transmission - Higher re-rupture rate (mainly in young people with high demand)
Peroneus longus	- Easy withdrawal technique - Less hypotrophy of the thigh - Larger graft diameter - Good functional results	- Donor site morbidity
Quadriceptal	- Possibility of use with or without bone plug - Modulus of elasticity similar to native LCA - Good functional results	- Donor site morbidity (residual pain and risk of patella fracture in cases of bone plug)

## Final Considerations

Patellar autograft with bone fragment remains the first choice for high-performance athletes who wish to return to their pre-injury sporting level, and flexor autograft is the first choice for patients with lower sporting demands. Allografts may be an alternative in patients with a lower level of physical activity, especially in those over 40 years of age. Quadriceptal and peroneus longus autografts have shown favorable functional results and are options of choice.

## References

[1] Herzog M M Marshall S W Lund J L Pate V Mack C D Spang J T Trends in Incidence of ACL Reconstruction and Concomitant Procedures Among Commercially Insured Individuals in the United States, 2002-2014 Sports Health 2018 10 06 523 531 30355175 10.1177/1941738118803616 PMC6204641

[2] Galatz L M Gerstenfeld L Heber-Katz E Rodeo S A Tendon regeneration and scar formation: The concept of scarless healing J Orthop Res 2015 33 06 823 831 25676657 10.1002/jor.22853 PMC6084432

[3] Bottoni C R Smith E L Shaha J Autograft versus allograft anterior cruciate ligament reconstruction: A prospective, randomized clinical study with a minimum 10-year follow-up Am J Sports Med 2015 43 10 2501 2509 26311445 10.1177/0363546515596406

[4] Shumborski S Salmon L J Monk C Heath E Roe J P Pinczewski L A Allograft donor characteristics significantly influence graft rupture after anterior cruciate ligament reconstruction in a young active population Am J Sports Med 2020 48 10 2401 2407 32736505 10.1177/0363546520938777

[5] Su M Jia X Zhang Z Medium-term (Least 5 Years) comparative outcomes in anterior cruciate ligament reconstruction using 4SHG, allograft, and LARS ligament Clin J Sport Med 2021 31 02 e101 e110 30855342 10.1097/JSM.0000000000000730 PMC7928216

[6] Goetz G de Villiers C Sadoghi P Geiger-Gritsch S Allograft for Anterior Cruciate Ligament Reconstruction (ACLR): a systematic review and meta-analysis of long-term comparative effectiveness and safety. results of a health technology assessment Arthrosc Sports Med Rehabil 2020 2 06 e873 e891 33376999 10.1016/j.asmr.2020.07.003 PMC7754611

[7] Guo L Yang L Duan X J Anterior cruciate ligament reconstruction with bone-patellar tendon-bone graft: comparison of autograft, fresh-frozen allograft, and γ-irradiated allograft Arthroscopy 2012 28 02 211 217 22244101 10.1016/j.arthro.2011.08.314

[8] MOON Knee Group Kaeding C C Pedroza A D Reinke E K Change in anterior cruciate ligament graft choice and outcomes over time Arthroscopy 2017 33 11 2007 2014 28847572 10.1016/j.arthro.2017.06.019 PMC5794339

[9] Sim K Rahardja R Zhu M Young S W Optimal graft choice in athletic patients with anterior cruciate ligament injuries: Review and clinical insights Open Access J Sports Med 2022 13 55 67 35800660 10.2147/OAJSM.S340702 PMC9255990

[10] Clark J C Rueff D E Indelicato P A Moser M Primary ACL reconstruction using allograft tissue Clin Sports Med 2009 28 02 223 244, viii 19306732 10.1016/j.csm.2008.10.005

[11] Krych A J Jackson J D Hoskin T L Dahm D L A meta-analysis of patellar tendon autograft versus patellar tendon allograft in anterior cruciate ligament reconstruction Arthroscopy 2008 24 03 292 298 18308180 10.1016/j.arthro.2007.08.029

[12] Kraeutler M J Bravman J T McCarty E C Bone-patellar tendon-bone autograft versus allograft in outcomes of anterior cruciate ligament reconstruction: a meta-analysis of 5182 patients Am J Sports Med 2013 41 10 2439 2448 23585484 10.1177/0363546513484127

[13] Kaeding C C Aros B Pedroza A Allograft versus autograft anterior cruciate ligament reconstruction: Predictors of failure from a MOON prospective longitudinal cohort Sports Health 2011 3 01 73 81 23015994 10.1177/1941738110386185 PMC3445196

[14] Barrett G R Luber K Replogle W H Manley J L Allograft anterior cruciate ligament reconstruction in the young, active patient: Tegner activity level and failure rate Arthroscopy 2010 26 12 1593 1601 20952145 10.1016/j.arthro.2010.05.014

[15] Borchers J R Pedroza A Kaeding C Activity level and graft type as risk factors for anterior cruciate ligament graft failure: a case-control study Am J Sports Med 2009 37 12 2362 2367 19684294 10.1177/0363546509340633

[16] Singhal M C Gardiner J R Johnson D L Failure of primary anterior cruciate ligament surgery using anterior tibialis allograft Arthroscopy 2007 23 05 469 475 17478276 10.1016/j.arthro.2006.12.010

[17] Lin K M Boyle C Marom N Marx R G Graft selection in anterior cruciate ligament reconstruction Sports Med Arthrosc Rev 2020 28 02 41 48 32345925 10.1097/JSA.0000000000000265

[18] Nagda S H Altobelli G G Bowdry K A Brewster C E Lombardo S J Cost analysis of outpatient anterior cruciate ligament reconstruction: autograft versus allograft Clin Orthop Relat Res 2010 468 05 1418 1422 20020337 10.1007/s11999-009-1178-y PMC2853669

[19] Park S S Dwyer T Congiusta F Whelan D B Theodoropoulos J Analysis of irradiation on the clinical effectiveness of allogenic tissue when used for primary anterior cruciate ligament reconstruction Am J Sports Med 2015 43 01 226 235 24477819 10.1177/0363546513518004

[20] Tian S Wang B Liu L Irradiated hamstring tendon allograft versus autograft for anatomic double-bundle anterior cruciate ligament reconstruction: Midterm clinical outcomes Am J Sports Med 2016 44 10 2579 2588 27466222 10.1177/0363546516655333

[21] Farago D Kozma B Kiss R M Different sterilization and disinfection methods used for human tendons - a systematic review using mechanical properties to evaluate tendon allografts BMC Musculoskelet Disord 2021 22 01 404 33941147 10.1186/s12891-021-04296-4 PMC8091719

[22] Legnani C Ventura A Terzaghi C Borgo E Albisetti W Anterior cruciate ligament reconstruction with synthetic grafts. A review of literature Int Orthop 2010 34 04 465 471 20157811 10.1007/s00264-010-0963-2 PMC2903133

[23] Satora W Królikowska A Czamara A Reichert P Synthetic grafts in the treatment of ruptured anterior cruciate ligament of the knee joint Polim Med 2017 47 01 55 59 29160630 10.17219/pim/76819

[24] Fan D Ma J Zhang L Patellar tendon versus artificial grafts in anterior cruciate ligament reconstruction: a systematic review and meta-analysis J Orthop Surg Res 2021 16 01 478 34348750 10.1186/s13018-021-02624-x PMC8336077

[25] Pan X Wen H Wang L Ge T Bone-patellar tendon-bone autograft versus LARS artificial ligament for anterior cruciate ligament reconstruction Eur J Orthop Surg Traumatol 2013 23 07 819 823 23412205 10.1007/s00590-012-1073-1

[26] Ventura A Terzaghi C Legnani C Borgo E Albisetti W Synthetic grafts for anterior cruciate ligament rupture: 19-year outcome study Knee 2010 17 02 108 113 19720536 10.1016/j.knee.2009.07.013

[27] Macaulay A A Perfetti D C Levine W N Anterior cruciate ligament graft choices Sports Health 2012 4 01 63 68 23016071 10.1177/1941738111409890 PMC3435898

[28] Machotka Z Scarborough I Duncan W Kumar S Perraton L Anterior cruciate ligament repair with LARS (ligament advanced reinforcement system): a systematic review Sports Med Arthrosc Rehabil Ther Technol 2010 2 29 21138589 10.1186/1758-2555-2-29 PMC3016369

[29] Bianchi N Sacchetti F Bottai V LARS versus hamstring tendon autograft in anterior cruciate ligament reconstruction: a single-centre, single surgeon retrospective study with 8 years of follow-up Eur J Orthop Surg Traumatol 2019 29 02 447 453 30232566 10.1007/s00590-018-2304-x

[30] Gao K Chen S Wang L Anterior cruciate ligament reconstruction with LARS artificial ligament: a multicenter study with 3- to 5-year follow-up Arthroscopy 2010 26 04 515 523 20362832 10.1016/j.arthro.2010.02.001

[31] Parchi P D Ciapini G Paglialunga C Anterior cruciate ligament reconstruction with LARS artificial ligament—clinical results after a long-term follow-up Joints 2018 6 02 75 79 30051101 10.1055/s-0038-1653950 PMC6059861

[32] Alvarez-Pinzon A M Barksdale L Krill M K Leo B M Hybrid graft anterior cruciate ligament reconstruction: a predictable graft for knee stabilization Orthopedics 2015 38 06 e473 e476 26091219 10.3928/01477447-20150603-54

[33] Kraeutler M J Kim S H Brown C C Clinical outcomes following primary anterior cruciate ligament reconstruction with hamstring autograft versus planned hybrid graft J Knee Surg 2018 31 09 827 833 29294500 10.1055/s-0037-1617417

[34] Vincelot-Chainard C Buisson X Taburet J F Djian P Robert H ACL autograft reconstruction revisions with tendon allografts: Possibilities and outcomes. A one-year follow-up of 39 patients Orthop Traumatol Surg Res 2022 108 03 102832 33556590 10.1016/j.otsr.2021.102832

[35] Hossain G MJ Islam M S Rahman Khan M M A prospective study of arthroscopic primary ACL reconstruction with ipsilateral peroneus longus tendon graft: Experience of 439 cases Medicine (Baltimore) 2023 102 09 e32943 36862908 10.1097/MD.0000000000032943 PMC9981376

[36] Rhatomy S Wicaksono F H Soekarno N R Setyawan R Primasara S Budhiparama N C Eversion and first ray plantarflexion muscle strength in anterior cruciate ligament reconstruction using a peroneus longus tendon graft Orthop J Sports Med 2019 7 09 2325967119872462 10.1177/2325967119872462 PMC6767728 31632995

[37] Rhatomy S Hartoko L Setyawan R Single bundle ACL reconstruction with peroneus longus tendon graft: 2-years follow-up J Clin Orthop Trauma 2020 11 03 S332 S336 32523289 10.1016/j.jcot.2019.09.004 PMC7275277

[38] Agarwal A Singh S Singh A Tewari P Comparison of functional outcomes of an anterior cruciate ligament (ACL) reconstruction using a peroneus longus graft as an alternative to the hamstring tendon graft Cureus 2023 15 04 e37273 37168157 10.7759/cureus.37273 PMC10164842

[39] Rhatomy S Asikin A IZ Wardani A E Rukmoyo T Lumban-Gaol I Budhiparama N C Peroneus longus autograft can be recommended as a superior graft to hamstring tendon in single-bundle ACL reconstruction Knee Surg Sports Traumatol Arthrosc 2019 27 11 3552 3559 30877316 10.1007/s00167-019-05455-w

[40] Wiradiputra A E Aryana G NW Peroneus longus tendon graft for anterior cruciate ligament reconstruction: A case report and review of literature Int J Surg Case Rep 2021 83 106028 34062359 10.1016/j.ijscr.2021.106028 PMC8178071

[41] Kerimoğlu S Aynaci O Saraçoğlu M Aydin H Turhan A U [Anterior cruciate ligament reconstruction with the peroneus longus tendon] Acta Orthop Traumatol Turc 2008 42 01 38 43 18354276 10.3944/aott.2008.038

[42] He J Tang Q Ernst S Peroneus longus tendon autograft has functional outcomes comparable to hamstring tendon autograft for anterior cruciate ligament reconstruction: a systematic review and meta-analysis Knee Surg Sports Traumatol Arthrosc 2021 29 09 2869 2879 32984919 10.1007/s00167-020-06279-9

[43] Kumar V K Narayanan S K Vishal R B A study on peroneus longus autograft for anterior cruciate ligament reconstruction Int J Res Med Sci 2020 8 01 183 188

[44] D'Ambrosi R Meena A Raj A Good results after treatment of RAMP lesions in association with ACL reconstruction: a systematic review Knee Surg Sports Traumatol Arthrosc 2023 31 01 358 371 35869982 10.1007/s00167-022-07067-3 PMC9859864

[45] Kusumastutia A H Rukmoyo T Rhatomy S Sakti Y M Anterior cruciate ligament reconstruction with peroneus longus tendon autograft: functional outcome and donor site morbidityOrthop J Sports Med 2020;8(5 Suppl 5):2325967120S00084

[46] Arora M Shukla T peroneus longus graft harvest: A technique note Indian J Orthop 2023 57 04 611 616 37006731 10.1007/s43465-023-00847-0 PMC10050498

[47] Kumar P M Shevte I Phalak M Nair A Arthroscopic anterior cruciate ligament reconstruction with semitendinosus graft versus peroneus longus tendon graft Int J Res Orthop 2020 6 02 386 392

[48] Angthong C Chernchujit B Apivatgaroon A Chaijenkit K Nualon P Suchao-in K The anterior cruciate ligament reconstruction with the peroneus longus tendon: A biomechanical and clinical evaluation of the donor ankle morbidity J Med Assoc Thai 2015 98 06 555 560 26219159

[49] Winkler P W Vivacqua T Thomassen S Quadriceps tendon autograft is becoming increasingly popular in revision ACL reconstruction Knee Surg Sports Traumatol Arthrosc 2022 30 01 149 160 33591370 10.1007/s00167-021-06478-y PMC8800889

[50] Lubowitz J H Editorial Commentary: Quadriceps tendon autograft use for anterior cruciate ligament reconstruction predicted to increase Arthroscopy 2016 32 01 76 77 26743412 10.1016/j.arthro.2015.11.004

[51] Meena A D'Ambrosi R Runer A Quadriceps tendon autograft with or without bone block have comparable clinical outcomes, complications and revision rate for ACL reconstruction: a systematic review Knee Surg Sports Traumatol Arthrosc 2023 31 06 2274 2288 36534150 10.1007/s00167-022-07281-z PMC10183433

[52] Kanakamedala A C de Sa D Obioha O A No difference between full thickness and partial thickness quadriceps tendon autografts in anterior cruciate ligament reconstruction: a systematic review Knee Surg Sports Traumatol Arthrosc 2019 27 01 105 116 29974173 10.1007/s00167-018-5042-z

[53] Kunze K N Moran J Polce E M Pareek A Strickland S M Williams R J III Lower donor site morbidity with hamstring and quadriceps tendon autograft compared with bone-patellar tendon-bone autograft after anterior cruciate ligament reconstruction: a systematic review and network meta-analysis of randomized controlled trials[published online ahead of print, 2023 Mar 31]Knee Surg Sports Traumatol Arthrosc 2023 31 08 3339 3352 10.1007/s00167-023-07402-2 37000243

[54] Runer A Csapo R Hepperger C Herbort M Hoser C Fink C Anterior cruciate ligament reconstructions with quadriceps tendon autograft result in lower graft rupture rates but similar patient-reported outcomes as compared with hamstring tendon autograft: A comparison of 875 patients Am J Sports Med 2020 48 09 2195 2204 32667271 10.1177/0363546520931829

[55] Özbek E A Dadoo S Chang A Rates of septic arthritis after ACL reconstruction: A single-center analysis highlighting quadriceps tendon grafts Am J Sports Med 2023 51 07 1708 1714 37092731 10.1177/03635465231165509

[56] Shani R H Umpierez E Nasert M Hiza E A Xerogeanes J Biomechanical comparison of quadriceps and patellar tendon grafts in anterior cruciate ligament reconstruction Arthroscopy 2016 32 01 71 75 26382635 10.1016/j.arthro.2015.06.051

[57] Woo S L Hollis J M Adams D J Lyon R M Takai S Tensile properties of the human femur-anterior cruciate ligament-tibia complex. The effects of specimen age and orientation Am J Sports Med 1991 19 03 217 225 1867330 10.1177/036354659101900303

[58] Urchek R Karas S Biomechanical Comparison of Quadriceps and 6-Strand Hamstring Tendon Grafts in Anterior Cruciate Ligament Reconstruction Orthop J Sports Med 2019 7 10 2325967119879113 10.1177/2325967119879113 PMC6801897 31667197

[59] Strauss M J Miles J W Kennedy M L Full thickness quadriceps tendon grafts with bone had similar material properties to bone-patellar tendon-bone and a four-strand semitendinosus grafts: a biomechanical study Knee Surg Sports Traumatol Arthrosc 2022 30 05 1786 1794 34591124 10.1007/s00167-021-06738-x

[60] Castile R M Jenkins M J Lake S P Brophy R H Microstructural and Mechanical Properties of Grafts Commonly Used for Cruciate Ligament Reconstruction J Bone Joint Surg Am 2020 102 22 1948 1955 32740264 10.2106/JBJS.19.01395

[61] ACL Study Group Arnold M P Calcei J G Vogel N ACL Study Group survey reveals the evolution of anterior cruciate ligament reconstruction graft choice over the past three decades Knee Surg Sports Traumatol Arthrosc 2021 29 11 3871 3876 33486558 10.1007/s00167-021-06443-9

