# Supplementary material for: New Graft Choices for ACL Reconstruction: Update Article
Source: Rev Bras Ortop (Sao Paulo). 2024 Apr 23;59(5):e642–9. doi: 10.1055/s-0044-1779335 (PMC11624934; doi:10.1055/s-0044-1779335)
Supplement: Related texts

# Research article: Novas técnicas de enxertia para reconstrucão do LCA: Artigo de atualização

**Keywords:** enxerto, joelho, ligamento cruzado anterior

## Abstract

A reconstrução do ligamento cruzado anterior (LCA) é um procedimento comum para lesões desse ligamento, especialmente em atletas. Existem diferentes tipos de enxertos utilizados, e a escolha depende de vários fatores. Os enxertos autólogos, do próprio paciente, são a opção mais comum, com rápida incorporação e menor taxa de falha. Enxertos aloenxertos, de doadores, têm seu papel em casos específicos. Os enxertos sintéticos, usados na década de 80, têm vantagens como ausência de morbidade no local doador, mas estudos mostraram complicações a longo prazo. Os enxertos híbridos, combinando enxertos autólogos e aloenxertos, têm ganhado interesse, permitindo um diâmetro maior e reduzindo a morbidade. O enxerto autólogo do tendão do músculo fibular longo tem recebido atenção, com resultados positivos, boa função do joelho e menor hipotrofia da coxa no local doador. O enxerto autólogo do tendão quadricipital tem ganhado popularidade, com resultados comparáveis aos enxertos de tendão patelar e de flexores, menor morbidade no local doador e menor taxa de re-ruptura. A escolha do enxerto evoluiu, com os enxertos autólogos de flexores sendo preferidos para pacientes menos ativos e o enxerto patelar com fragmento ósseo para atletas de alta performance. Enxertos aloenxertos, sintéticos e híbridos têm seu papel em circunstâncias específicas. A escolha deve ser baseada em evidências científicas, considerando vantagens e desvantagens. A reconstrução do LCA é um procedimento complexo que requer considerações individuais para selecionar o enxerto mais adequado.

## Introdução

A ruptura do ligamento cruzado anterior (LCA) é uma lesão frequente na população em geral, com uma incidência de até 75 por 100.000 pessoas por ano, 1 particularmente em indivíduos ativos envolvidos em esportes de contato. Embora um LCA reconstruído não restabeleça completamente a estrutura original ou propriedades biomecânicas do LCA nativo, 2 o enxerto usado para a reconstrução não deve apenas ter propriedades estruturais e mecânicas que se assemelham àquelas do ligamento nativo, deve também apresentar antigenicidade mínima e potencial biológico inato suficiente para incorporar ao osso do hospedeiro. Ao selecionar os tipos de enxerto, há várias considerações: autoenxerto versus aloenxerto e enxertos de partes moles apenas versus enxertos com fragmentos ósseos. Na Fig. 1 são demonstrados exemplos de aloenxertos.

**Fig. 1:** Foto de aloenxertos prontos para serem preparados. Observam-se enxerto com parte óssea (Patelar) e sem partes ósseas (Tibial Posterior).

Os autoenxertos comumente utilizados são: patelar com fragmento ósseo, flexores do joelho, quadríceps (com ou sem fragmento ósseo patelar); entre os aloenxertos, opções adicionais incluem tibial anterior e posterior, fibular e calcâneo. 3 4 5 6 7 Na Fig. 2 são demonstrados diferentes tipos de enxertos.

**Fig. 2:** Diferentes tipos de enxertos: (A) enxerto do tendão patelar, (B) enxerto do tendão quadriciptal com plug ósseo, (C) enxerto sêxtuplo dos flexores.

A seleção ideal do enxerto depende não apenas das propriedades do enxerto, mas principalmente das características e expectativas do paciente.

## Autoenxerto Versus Aloenxerto

Todos os aloenxertos demonstraram taxas mais lentas de incorporação comparados aos autoenxertos, além de uma taxa mais alta de cerca de 25% de falha na população ativa (43 versus 75%). 8 Evidência atual sugere o uso de aloenxertos em circunstâncias específicas como reconstruções multiligamentares do joelho, tecido inadequado de autoenxerto ou em populações mais idosas e menos ativas. 9 As vantagens teóricas dos aloenxertos são: eliminação da morbidade do sítio doador, menos dor, tempos cirúrgico e de reabilitação menores e melhor resultado cosmético. 10 Krych et al. 11 relataram um risco cinco vezes maior de reruptura em casos que utilizaram aloenxerto. Ao excluir enxertos irradiados e quimicamente processados, não houve diferença na taxa de reruptura; porém a revisão sistemática deles incluiu apenas 6 estudos. Kraeutler et al. 12 demonstraram resultados semelhantes com um risco de reruptura cerca de 3 vezes maior no grupo do aloenxerto (12,7% vs. 4,3%). Também demonstraram aumento da frouxidão do joelho, piores resultados no teste *single-leg hop* e na satisfação subjetiva. 12

O estudo de coorte prospectivo realizado por Kaeding et al. 13 avaliou o número de variáveis para determinar preditores da ruptura do enxerto nos 2 anos após a reconstrução. Uso de aloenxerto e idade jovem aumentaram significativamente o risco de ruptura do enxerto. 13 Outros estudos recentes encontraram taxas aumentadas de ruptura do enxerto em pacientes que receberam aloenxertos e tiveram um nível alto de atividades no pós-operatório. 14 15 16

Em estudos em laboratórios e clínicos os autoenxertos apresentam resultados melhores do que os aloenxertos irradiados e processados. 17 Aloenxertos podem ser considerados para pacientes menos ativos que estão dispostos a aceitar o risco aumentado de falha do enxerto. 17

Em um ambiente de cirurgia ambulatorial, o grupo do autoenxerto teve uma conta significativamente menor do que o grupo do aloenxerto. 18 O decrécimo do tempo cirúrgico não compensou o custo do aloenxerto (cerca de $1000). 18

Em relação ao processamento do enxerto acredita-se que as técnicas de esterilização alteram as propriedades biomecânicas e quanto mais um enxerto é processado, pior é seu desempenho. 17 Park et al. 19 realizaram uma revisão sistemática de aloenxertos irradiados versus não irradiados com pelo menos dois anos de seguimento, demonstrando no grupo irradiado piores escores funcionais, estabilidade diminuída para Lachman, pivot-shift e teste no KT-1000 e risco aumentado de revisão. O estudo de Tian et al. 20 demonstrou resultados semelhantes. Técnicas de esterilização do aloenxerto alteram as propriedades mecânicas dos aloenxertos e são categorizadas em radiação ou óxido de etileno. A extensão da alteração das propriedades mecânicas é dependente da dose de exposição de irradiação. 7

Farago et al. 21 revisaram artigos de 29 anos que avaliaram o impacto das técnicas de esterilização nos tendões. Resultados da revisão suportam que a técnica com maior preservação biomecânica foi o congelamento seguido de radiação a 14.8-28.5 kGy. 21 Entretanto, falha do aloenxerto não é atribuida apenas às técnicas de esterilização, ao passo que taxas de falha do aloenxerto ainda continuam alta quando comparados aloenxertos frescos a autoenxertos. 21

## Enxertos Sintéticos

Foram utilizados como opção de enxerto inicialmente na década de 80, com as vantagens de ausência de morbidade do sítio doador, tempo cirúrgico menor e risco reduzido de transmissão de doenças. 22 23 24 E potencialmente uma possibilidade de um retorno mais precoce aos esportes. 22 23 24 Entretanto, estudos iniciais reportaram resultados satisfatórios a curto prazo mas a médio e longo prazo houve complicações como resposta imunológica, sinovite por corpo estranho, osteólise dos túneis, fraturas do fêmur e tíbia perto dos túneis e falha tardia do enxerto. 22 23 24 25 26 Isso resultou em um declínio no uso de enxertos sintéticos, porém há novamente um interesse atual em uma nova geração de enxertos artificiais que demonstraram resultados favoráveis quando utilizados em circunstâncias especiais como na população mais idosa. 24 25 Uso tem ganhado certa popularidade entre atletas recentemente devido potencial para estabilidade imediata do enxerto, reabilitação mais rápida e um retorno mais rápido ao esporte. 27 Revisão sistemática realizada por Machotka et al. 28 sobre o *Ligament Advanced Reinforcement System* recomendou atenção ao considerar o uso de enxerto sintético visto que mais estudos são necessários. No estudo de Bianchi et al. 29 que comparou LARS e enxerto de flexores do joelho foi demonstrado que o grupo LARS apresentou uma estabilidade maior e em nenhum paciente foi necessária uma cirurgia de revisão. LARS pode ser considerado em pacientes que necessitam uma recuperação rápida ciente do risco de falha do enxerto e osteartrose iatrogênica. 30 31

## Enxerto Híbrido

Consiste da combinação de auto e aloenxertos e foi inicialmente descrito em 2015. 32 Esses enxertos, tipicamente formados de uma combinação de enxerto autólogo de flexores com aloenxerto de partes moles, ganharam interesse dos ortopedistas para uso em reconstruções de LCA. 33 São utilizados geralmente em casos de tamanho pequeno dos enxertos de flexores. 32 Ao planejar usar enxertos híbridos, pode-se retirar apenas o enxerto do semitendíneo ao invés do semitendíneo e grácil. Com isso, o uso de enxertos híbridos pode reduzir a morbidade pós-operatória do local doador. 33 Geralmente utiliza-se um aloenxerto do tibial posterior ou fibular longo não irradiado. Além do benefício da menor morbidade do local doador, os enxertos híbridos também permitem que o enxerto tenha um diâmetro maior do que o autoenxerto semitendíneo/grácil, o que pode reduzir o risco de falha pós-operatória. Com isso, enxertos híbridos podem ser uma opção para pacientes mais idosos. 33

## Enxerto Autólogo do Tendão do Músculo Fibular Longo

Como enxerto autólogo, o tendão do músculo fibular longo é uma opção antiga, entretanto tem recebido uma atenção maior nos últimos anos devido às suas propriedades biomecânicas semelhantes ao ligamento nativo do LCA e ao enxerto dos isquiotibiais. 34 35 36 Na Fig. 3 são demonstrados o enxerto e sua retirada.

**Fig. 3:** Enxerto do Fibular curto: (A) pequena via de acesso para retirada do enxerto com mínima morbidade e (B) enxerto do tendão fibular curto pronto para ser preparado na mesa com bom comprimento e boa espessura.

Estudos mais recentes avaliaram o seu uso na reconstrução do LCA e bem como a função pós-operatória e a morbidade no local doador. Esses estudos consistentemente demonstram resultados positivos, apoiando a viabilidade do enxerto autólogo do tendão do músculo fibular longo como uma boa opção de enxerto nas reconstruções. 37 38 39 40

Em termos de resultados funcionais, os pacientes submetidos à reconstrução do LCA usando o enxerto autólogo do tendão do músculo fibular longo obtiveram excelentes pontuações em várias ferramentas de avaliação, sendo as principais os escores IKDC, Modified Cincinnati, Tegner-Lysholm, AOFAS e FADI. 35 41 42

Essas pontuações indicam boa função do joelho, estabilidade do tornozelo e satisfação geral do paciente. Também foram obtidos resultados funcionais comparáveis às opções tradicionais de enxerto, como o enxerto autólogo do tendão dos isquiotibiais. 35 43 44 Além disso, o enxerto autólogo do tendão do músculo fibular longo demonstrou vantagens em relação a outras opções de enxerto. Ele apresentou um diâmetro de enxerto maior, o que pode contribuir para propriedades mecânicas e estabilidade aprimoradas. 35 36 39 45 Foi associado a uma menor hipotrofia da coxa, indicando uma redução na perda muscular no local doador. A atividade do tornozelo do doador não foi comprometida, conforme evidenciado por pontuações positivas em ferramentas de avaliação da função do tornozelo e testes de salto. 35 36 40

Outro fator que torna o tendão do músculo fibular longo um candidato ideal para a reconstrução do LCA é de ser tecnicamente seguro e fácil sua retirada. 46 O tendão está superficialmente localizado e sua posição não é complicada de ser acessada por estruturas adjacentes, como os tendões do músculo posterior da coxa. 46 47

A morbidade no local doador foi mínima, não sendo observadas diferenças significativas na eversão do tornozelo e na força de plantiflexão do primeiro raio entre o local doador e o local saudável contralateral. 35 36 Esses achados sugerem que o enxerto autólogo do tendão do músculo fibular longo não causa morbidade significativa no local doador. 48

Em conclusão, a reconstrução do LCA usando o enxerto autólogo do tendão do músculo fibular longo é um procedimento cientificamente respaldado, com resultados favoráveis. Ele oferece resultados funcionais comparáveis às opções tradicionais de enxerto, como o tendão dos isquiotibiais e pode ser utilizado como alternativa aos enxertos autólogos mais comumente utilizados para a reconstrução do LCA; o tendão patelar e o tendão do músculo posterior da coxa.

## Enxerto Autólogo do Tendão Quadricipital

O enxerto autólogo do tendão quadricipital (TQ) para reconstrução do LCA, apesar de ser atualmente ainda um dos menos utilizado dos enxertos, apresenta um aumento na sua popularidade nos últimos anos, tanto para reconstruções primárias como para revisões. 49 50 O enxerto TQ pode ser utilizado com ou sem fragmento ósseo retirado da patela. As vantagens da retirada do enxerto TQ com fragmento ósseo são um enxerto com maior comprimento e uma possível melhor integração da parte óssea do enxerto no túnel confeccionado para o enxerto. As desvantagens são uma possível dor residual no local de retirada do bloco ósseo patelar e o risco de fratura da patela. Apesar dessas diferenças, uma revisão sistemática recente comparou a utilização do enxerto TQ com ou sem fragmento ósseo e mostrou que ambos os enxertos são seguros e viáveis, com resultados clínicos, complicações e taxa de revisão comparáveis. 51 Além disso, o enxerto TQ pode ser utilizado com espessura parcial ou total, sem diferença entre eles em revisão sistemática recente. 52

Quando comparado às demais opções de enxerto, o enxerto TQ possui benefícios como menor morbidade no sítio doador (definido como dor anterior do joelho, dificuldade ou impossibilidade para ajoelhar-se ou combinados) quando comparado ao enxerto autólogo de tendão patellar. 53 Em estudo de coorte, a reconstrução do LCA com o uso de enxerto TQ apresentou menor taxa de re-ruptura quando comparado ao enxerto autólogo de flexores. 54 Em estudo recente com 6652 reconstruções de LCA, os casos com utilização do enxerto TQ apresentaram menor taxa de artrite séptica quando comparados aos casos que utilizaram enxerto de flexores, de tendão patelar ou aloenxertos. 55 Em relação à biomecânica, o enxerto TQ apresenta módulo de elasticidade similar ao do LCA nativo, o que, pelo menos do ponto de vista teórico, é positivo pois permitiria uma biomecânica mais próxima da biomecânica do joelho antes da lesão. 56 57 Enquanto isso, tanto o enxerto de tendão patelar quanto o enxerto de flexores possuem módulo de elasticidade significativamente maior do que o do LCA nativo. 57 58 Já quanto a carga para falha, o enxerto TQ apresenta uma carga semelhante a carga do enxerto de flexores e uma carga significativamente maior do que as cargas do enxerto de tendão patelar e do LCA native. 56 57 58 59 60 Por fim, em relação aos resultados clínicos de taxa de ruptura de enxerto e de desfechos reportados pelos pacientes, a literatura atual não apresenta diferenças significativas quando o enxerto TQ é comparado aos enxertos de tendão patelar e de flexores. 61

## Discussão

O estudo de Arnold et al. 61 demonstrou o resultado de pesquisa realizada nos últimos 14 encontros do ACL group sobre qual tipo de enxerto é a preferência dos cirurgiões em reconstruções do LCA. Ao longo do tempo a escolha do tipo de enxerto pode ser dividida em 4 fases: dominância do enxerto autólogo patelar com fragmento ósseo; dominância do enxerto autólogo patelar com fragmento ósseo com aumento do enxerto autologo de flexores; dominância do enxerto autólogo de flexores com diminuição do enxerto autólogo patelar com fragmento ósseo e aumento do uso de aloenxertos e finalmente dominância do enxerto autólogo de flexores com manutenção dos níveis de escolha dos flexores do joelho e aumento do enxerto autólogo quadriceptal. Atualmente mais de 50% dos entrevistados referem que a primeira escolha é o enxerto autólogo de flexores, enquanto menos de 40% utilizam enxerto autólogo de patelar com fragmento ósseo. Aloenxertos aumentaram em popularidade de 2006 a 2012, alcançando 12% das escolhas em 2012. Atualmente, apenas 1% dos entrevistados utilizam aloenxertos como primeira escolha e nenhum utliza aloenxerto com fragmento ósseo. Autoenxerto quadriceptal tem aumentado em frequência de escolha desde 2014 com um pico acima de 10% em 2018. 61

Ao selecionar um enxerto para uma reconstrução primária do LCA devemos levar em consideração uma série de fatores, incluindo idade, nível de atividade, lesōes prévias, entre outros. Cada opcão de enxerto possui suas vantagens e desvantagens, Os autoenxertos patelar e de flexores do joelho ainda são os mais utilizados mas há outras opcoes conforme mostrado na parte inicial deste artigo. Aloenxertos, autoenxerto quadricipital e do fibular longo são opcoes e suas principais vantagens e desvantagens são mostradas na Tabela 1 .

**Tabela 1:** Vantagens e desvantagens dos enxertos citados

Tipos de enxerto	Vantagens	Desvantagens
Aloenxerto	- Ausência de morbidade do local doador - Escolha do tamanho do enxerto - Menor tempo cirúrgico - Mlehor resultado cosmético	- Alto Custo - Baixa disponibilidade - Risco de transmissão de doenças - Maior taxa de reruptura (principalmente em jovens com alta demanda)
Fibular longo	- Técnica de retirada fácil - Menor hipotrofia da coxa - Diâmetro maior do enxerto - Bons resultados funcionais	- Morbidade do local doador
Quadriceptal	- Possibilidade de uso com ou sem plug ósseo - Módulo de elasticidade semelhante ao LCA native - Bonsresultados funcionais	- Morbidade de local doador (dor residual e risco de fratura de patella nos casos de plug ósseo)

## Considerações Finais

O autoenxerto patelar com fragmento ósseo permanece como a primeira escolha para atletas de alta performance que desejam retornar ao nível esportivo pré-lesão e o autoenxerto de flexores é a primeira escolha para pacientes com uma demanda esportiva menor. Aloenxertos podem ser uma alternativa em pacientes com menor nível de atividade física, especialmente naqueles acima de 40 anos. Os autoenxertos quadriceptal e do fibular longo mostraram resultados funcionais favoráveis e são opções de escolha.
